# Solid organ transplantation originating from uncontrolled donation after circulatory death in Europe: a narrative review

**DOI:** 10.1186/s13049-024-01305-y

**Published:** 2024-12-18

**Authors:** Yann Pionnier, Tom Darius, Andrea Penaloza, Francoise Steenebruggen, Florence Dupriez, Arne Neyrinck, Cornelia Genbrugge

**Affiliations:** 1 Emergency Department, Cliniques Universitaires Saint-Luc, Brussels, Belgium; 2 Department of Abdominal Surgery and Transplantation, Cliniques Universitaires Saint-Luc, Brussels, Belgium; 3 Department of Anesthesiology, University Hospitals Leuven, Louvain, Belgium; 4 Emergency Department, Cliniques Universitaires Saint-Luc, Emergency Medicine, Department of Public Health and Primary Care, Faculty of Medicine, Catholic University Leuven, Brussels, Belgium

**Keywords:** Narrative review, Organ transplantation, Organ shortage, Controlled donation after circulatory death (cDCD), Uncontrolled donation after circulatory death (uDCD), Out-of hospital cardiac arrest, Extracorporeal Circulation, Ethics, Economics

## Abstract

Human organ transplantation has begun in the 1960s with donation after circulatory death. At that time this was named non heart beating donation, later donation after cardiac death and nowadays it is named donation after circulatory death. Currently, we are facing a significant shortage of transplant organs in Europe and worldwide. To increase the graft acceptance from donation after controlled or uncontrolled circulatory death, preceding regional normothermic perfusion by an extracorporeal circulation before organ procurement or ex-situ machine perfusion are frequently implemented in clinical practice as organ assessment and reconditioning techniques. Due to these advancements more organs can be potentially transplanted, even after out-of-hospital cardiac arrest (OHCA). First line actors like emergency physicians and pre-hospital paramedics must be aware of such programs to recognize and refer patients for donation in OHCA situations. This review provides an overview of organs transplanted from uncontrolled donation after circulatory death (uDCD) and emphasize the role of the emergency physician in the organ donation cascade. Outcome of uDCD has a lower effectiveness than donation after brain death (DBD) and controlled donation after circulatory death (cDCD) for short term graft survival. However, observational studies illustrate that long term outcome from uDCD is comparable to graft outcome from cDCD and DBD. We summarize the studies reporting the procured organ rate and functional outcome of organs originated from uDCD. European databases indicate a high incidence of OHCA, where resuscitation efforts are initiated but the rate of return of spontaneous circulation (ROSC) remains limited. These patients represent a substantial potential pool of organ donors for uDCD programs. However, these programs tend to overestimate the number of potential donors. While organ procurement from uDCD has yielded favorable outcomes, further research is required to accurately assess the associated costs and benefits and to establish clear donor selection guidelines. Furthermore, the use of new technologies like extracorporeal Cardiopulmonary Resuscitation (E-CPR) for organ donation should be investigated from both medical and economical perspectives. Emergency departments must also explore the feasibility of implementing these programs.

## Introduction

Human organ transplantation started in the 1960s with non-heart beating donation, later called donation after cardiac death and at present called donation after circulatory death [1]. Nowadays we are facing a significant shortage of donor organs in Europe and worldwide which is fuelled by a higher rate of end stage diseases. In the last decade, in order to face the shortage of organs, organ donation after controlled and uncontrolled circulatory death are reconsidered to increase the donation pool with advanced technologies like extracorporeal circulation and/or ex-situ conservation. The EuReCa study identified 25,171 cases of out-of-hospital cardiac arrest (OHCA) in which resuscitation started, in 27 European countries during a three month period [2]. Return of spontaneous circulation (ROSC) occurred in 32.7% of these patients, revealing a missed potential for uDCD from refractory cardiac arrest (CA). Retrospective studies examining this patient population and applying organ donation criteria, different between countries, have found that the proportion of OHCA patients eligible for uDCD ranged from 4.3 to 19.6% [3–5]. However, these are retrospective studies, as practical aspects, such as the need for advanced resources required for extracorporeal cardiopulmonary resuscitation (eCPR), are taken into account this potential number should be decreased but currently no study was performed to calculate the missed opportunities. In reality, only a limited number of European countries have introduced uDCD programs (Maastricht Category II) and many of these have limited uDCD activity with the quantitatively most developed programs in France and Spain [6]. As time is a critical factor, especially in the uncontrolled donation after circulatory death (uDCD), early recognition and activation of the chain of the organ donation cascade is of utmost importance in which the emergency physician plays a key role.

This review will focus on uncontrolled donation after circulatory death in Europe and the potential to decrease the transplantation waiting list.

### Donation after circulatory death (DCD)

**Fig. 1:**
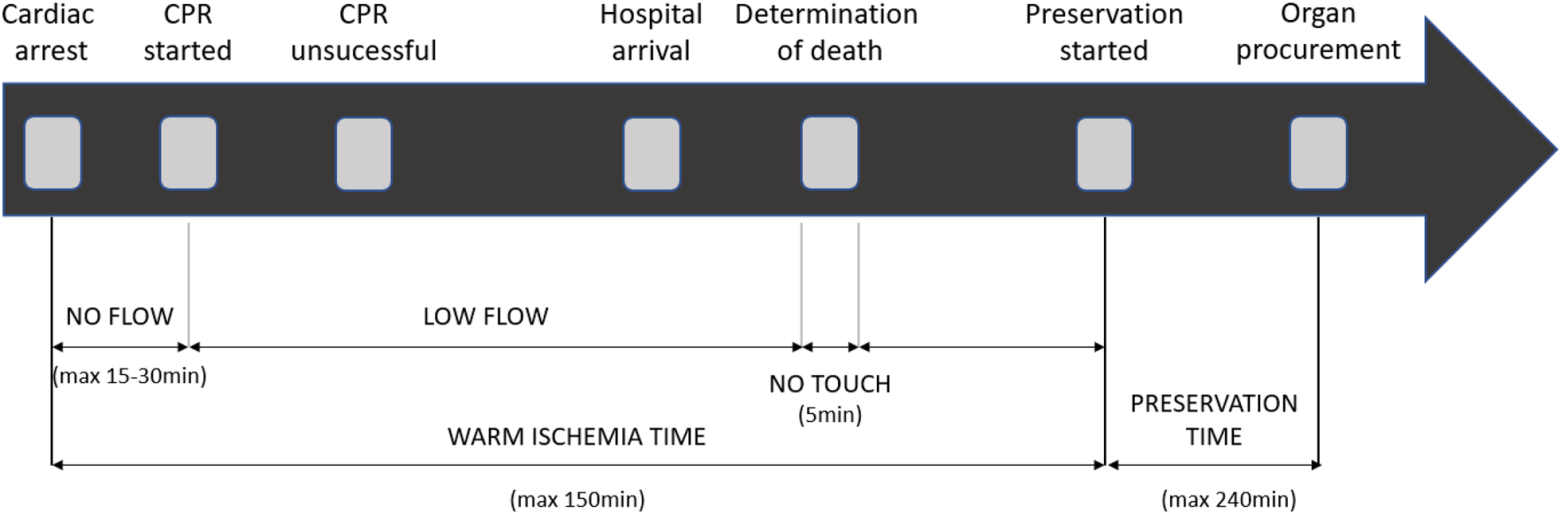
Process of uncontrolled donation after circulatory death

The modified Maastricht Classification defines five different categories for organ donation after circulatory death (Fig. 1). Category I is an unwitnessed death without any attempt of resuscitation. This category is currently not used in Belgium nor Europe as it is legally prohibited to transport a deceased person by ambulance. Category II is a witnessed cardiac arrest (CA) with unsuccessful resuscitation. These two groups are unexpected so defined as uncontrolled (uDCD) and are subdivided into A and B to describe out or in-hospital CA. This subdivision was added because the outcome is weaker in the OHCA patients due to increase of warm ischemia time and logistics [7]. Category III is death following withdrawal of life-sustaining therapies (WLST) so defined as controlled (cDCD). Category IV refers to an unexpected CA after determination of brain death. In this scenario, resuscitation may be performed. If return of spontaneous circulation (ROSC) is not achieved the patient is considered as potential uDCD donation. cDCD could also been performed in this category when the CA is highly expected and happen in the operating theatre or intensive care. Category V is organ donation after euthanasia. The terminology uncontrolled and controlled was added with the objective to distinguish the ischemic times [7] (Table 1). Currently, the Maastricht category III is the largest source for procurement of organs for donation.

**Table 1:** The modified Maastricht classification

Categories	Subcategories	Description
*Category I (Found dead)*		
Uncontrolled	IA: Out of hospital	Sudden unexpected CA without any attempt in resuscitation
*Category II (witnessed CA)*		
Uncontrolled	IB: In hospital	
	IIA: Out of hospital	Sudden unexpected irreversible CA with unsuccessful resuscitation
	IIB: In hospital	
*Category III (WLST)*		
Controlled		Planned withdrawal of life-sustaining therapy, expected CA
*Category IV*		
Uncontrolled controlled		Sudden CA after brain death diagnosis during donor life- management
*Category V*		
Euthanasia		Planned euthanasia

*CA* cardiac arrest, *WSLT* withdrawing life sustaining therapies

All DCD procedures follow a process leading to the diagnosis of cardiac death, though significant logistical differences exist between uncontrolled (uDCD) and controlled DCD (cDCD). This process can be delineated into distinct time periods. The first period is total warm ischemia time (WIT), defined as the interval from withdrawal of life-sustaining therapy (WLST) to organ preservation in cDCD, or from cardiac arrest (CA) to organ preservation in uDCD. In uDCD, WIT is further subdivided into absolute WIT and functional WIT. Absolute WIT refers to the “no-flow” time from cardiac arrest to the initiation of CPR, while functional WIT spans from the start of CPR to the beginning of cannulation in uDCD, encompassing the “no-touch” period (Fig. 1).

In cDCD, functional WIT is defined as the period from the point at which systolic blood pressure falls to 50–60 mmHg until the cooling technique is initiated, also including the no-touch period. Total WIT in cDCD sequentially begins at WLST and concludes with the initiation of the cooling technique. However, logistical demands and time management differ considerably between uDCD and cDCD, as uDCD involves unanticipated events requiring rapid decision-making and preparation within narrow timeframes.

This is followed by the cold ischemia time (CIT) which is the time between organ preservation and the grafting. Both, the WIT and CIT are well-known factors to increase graft complication [8].

At last, the “no touch” period is defined as the time between the cessation of circulation and respiration and the determination of death and is literally a period where the patient is not touched even not for non-therapeutic purposes [7]. Currently, it is accepted that the no touch period should be interpreted as the time needed to define death based on permanent cessation of vital functions. In practice, the no touch period is installed to observe the absence of autoresuscitation. This no touch period is by law mandatory (Fig. 1) but varies between different countries going from 3 to 5 min in Belgium to 20 min in Italy [6, 9]. It is important to acknowledge these critical time periods and define time to be able to ameliorate the procedure to improve organs quality [8, 10].

### Uncontrolled donation after circulatory death (uDCD)

#### uDCD procedure and key steps

An initial search was conducted on PubMed using the terms “organ transplantation,” “uncontrolled donation after circulatory death,” “cardiac arrest,” “extracorporeal circulation,” and “outcomes.” All English and French articles from 2000 to 2023 were included. Abstracts were initially screened to make a preliminary selection. The selected references were reviewed by the first author, who identified articles relevant to the topic. Additional searches were conducted within European transplantation databases, specifically Agence de la Biomédecine for France, Eurotransplant, Organización Nacional de Trasplantes (ONT), as well as in ERC guidelines. The category IIA (OHCA) or IIB (IHCA) donation starts when an emergency medical service attends a witnessed, unexpected CA in a patient in whom resuscitation does not lead to return of spontaneous resuscitation (ROSC) despite advanced life support (ALS). To this day, different criteria are used in different centers even in the same country, to select potential organ donors who have undergone CA (Table 2) [11, 12]. The ILCOR published a basic protocol to create a uDCD program but noticed a lack of evidence to create an international guideline. This lack of evidence may be attributed to differing legal frameworks across countries, and sometimes even between regions, necessitating the development of local protocols. Consequently, there is insufficient comparable data to support broader generalizations. [11, 13, 14]. As a result, each center is trying to find the optimal criteria for their institution, taking into account the efforts versus the benefits for the local situation [11, 14]. The declaration of death is made by the physician responsible for the patient and is independent of the transplant team to ensure that death is not declared while therapeutic options remain viable. To address ethical considerations, the World Health Organization has published guidelines on this process [13]. The whole process of uDCD is resumed in Fig. 1.

**Table 2:** Criteria for uDCD

Criteria for uDCD
> 18 years
< 60 years
Known of suspected causes of cardiac arrest
No-flow < 15 min
Transport time < 90 min
Registered for organ donor
No exclusion criteria for organ donation

Following the confirmation of death, protocols in France and Spain permit the re-establishment of cardiac compression and mechanical ventilation for the sole purpose of organ preservation. However, this practice is not permitted in the Netherlands. This is allowable as death is confirmed following exhaustive advanced CPR, characterized by prolonged no-flow and low-flow periods [15].

After the confirmation of death and the no touch period, the transplantation team will start organ reconditioning techniques preceding the procurement procedure itself. Such techniques consist either of in-situ cooling using a double-balloon-triple lumen catheter technique, or the establishment of hypothermic or normothermic regional perfusion (nRP) of organs. In-situ preservation strategies provide time to complete consent, authorization requirements, evaluate the individual’s suitability for donation and organize the procurement team [13, 16].

Kidneys, liver, pancreas and even lungs are organs which can be potentially procured during uDCD [17, 18]. However, the majority of transplanted organs are kidneys and liver in uDCD, due to logistical difficulties to start preservation within an acceptable delay for other organs. The practical implementation of a uDCD at the emergency department is limited by the critical WIT and the localisation of the OHCA. In uDCD WIT is prolonged with the no flow time, resuscitation time and transport time. Warm ischemia time can be reduced thanks to strict criteria resumed in Table 2. However, not all criteria are known in the pre-hospital setting when the decision is made to transport a patient with ongoing CPR to the hospital for potential organ donation. This poses not only logistical challenges but also ethical and financial ones. In these urgent and unpredictable situations, the pre-hospital physician must consider the possibility of organ donation despite lacking the patient's medical history and wishes. Given the critical time constraints, the decision to transport the patient and activate the procurement team must be made rapidly. Beyond logistical issues, managing these cases from an ethical perspective is also challenging, as initiating discussions about organ donation, if family members are present, may not always be appropriate. Therefore, the role of the emergency physician is crucial, as they act as the primary gatekeeper in this process. This factor also contributed to the low numbers of uDCD cases in the Dutch databases reported by Brat et al. [19], as eligible patients were declared dead pre-hospital without transfer to the emergency department, as we know that 50% of OHCA patients are declared death pre-hospitally [13]. Moreover, these strict criteria can also limit the potential of organ donors, which can minimize the benefit of an uDCD program [19]. This highlights once again the important role of the pre-hospital emergency physician and emergency team in recognizing the potential for uDCD and involving them in the development of a uDCD program. Maximum of WIT of 150 min is used in France and Spain [20] and can be optimized by performing good quality CPR [6, 19]. Damage due to CIT is limited because of all recent innovative research about preservation techniques which can reduce the impact of CIT on grafts [21, 22]. Compared to in-situ cooling, nRP is associated with significantly improved graft function at two years post-transplantation even with livers and lungs transplanted from uDCD [23, 24].

#### Outcomes

Between 16.9 and 73.9% of initiated procedures for uDCD result in actual organ donors. (Table 3) [25]. The uDCD donation has a lower effectiveness than DBD and cDCD for short term graft survival [6, 26–28]. This suggests that the selection of organs is fundamental and more research is necessary to phenotype the ideal donor in uDCD. The lower effectiveness, together with the practical difficulties and strict inclusion criteria could also explain why uDCD programs are only available in a few countries, next to the ethical burden. Concerns have been raised regarding the quality of procured organs, with the perception that the efforts involved may not justify the benefits. However, there is a lack of research examining the economic burden associated with the activation of the transplant team in uDCD, as well as the long-term function of the transplanted organs. Moreover, observational studies show that long term uDCD graft success is comparable to other organ procurement [29–34]. Long‐term survival and graft function were comparable between recipients from uDCD and DBD donors [22]. However, kidney transplants from uDCD have a higher incidence of primary non-function and delayed graft function compared with DBD and cDCD organs [22, 30]. Data from France in 2015, show 88.9% 5-year graft survival for kidneys in DBD [35], compared to 63% and 60% in uDCD studies [36, 37]. Table 3 shows an overview of studies, conducted between 2000 and 2023, reporting on uCDCD, the actual procured organs and the functional outcome of the organs. Comparing graft effectiveness between uDCD and other procurement method is difficult due to differences in legal frameworks, type of preservation used, and selection criteria. Moreover, in the literature uDCD do not always go through the same process, as sometimes ECMO is used as bridge between advanced CPR and organ procurement. Between 31.5 and 73.9% of potential uDCD donors become actual donors. However, the rate of primary non-function ranges from 5.1 to 27%, with a five-year graft survival rate of 60% (Table 3).

**Table 3:** Review of uDCD studies

Authors, Date, Country	Type of studies, Date of the study	Patient included, Organs procured	Inclusion criteria uDCD	Outcomes
De Antonio et al., 2007, Spain	Retrospective	54,	*Absence neoplasia, systemic diseases	*31,5% effective donors
	2002–2006	Lungs	*1–55 years	*17 lungs transplanted
			*Witnessed CA	*53% PNF
			*No flow < 15 min	
			*WIT < 120 min	
			*No traumatic massive bleeding	
Mateos-Rodriguez et al.,	Retrospective	28,	*Absence neoplasia, systemic diseases	*39 kidneys transplanted
2010, Spain	January 2008–April 2009	Kidneys	*1—55 years	*5,1% PNF
			*Witnessed CA	
			*No flow < 15 min	
			*Arrival hospital < 90 min after CA	
			*No traumatic massive bleeding	
Fondevila et al.,	Retrospective	290,	*Absence neoplasia, systemic diseases	*50% effective donors
2012, Spain	April 2002–December 2010	Liver	*1—65 years	*34 livers transplantation
			*Witnessed CA	*82% 1 year graft survival
			*No flow < 15 min	
			*Arrival hospital < 90 min after CA	
			*No traumatic massive bleeding	
Mateos-Rodriguez et al.,	Retrospective	214,	*Absence neoplasia, systemic diseases	*73,9% effective donors
2012, Spain	January 2005–April 2010	Kidneys, Liver and Lungs	*1—55 years	*302 organs transplanted
			*Witnessed CA	*Functionality rate: 91% for kidneys, 75% for livers
			*No flow < 15 min	
			*Arrival hospital < 90 min after CA	
			*No traumatic massive bleeding	
Hoogland et al.,	Retrospective	Unknown,	*Absence neoplasia, systemic diseases	*83 effective donors
2011, Nederlands	January 1981–January 2008	Kidneys	* < 65 years	*138 kidneys transplanted
			*Witnessed CA	*22% PNF
			*CPR < 45 min (< 90 min in donors < 50 years)	*63% 5 year graft survival
			*Time between cessation of resuscitation and start of in situ preservation < 45 min	
Peters-Sangers et al.,	Retrospective	133,	*Absence neoplasia, systemic diseases	*49,6% effective donors
2017, Nederlands	January 2002–January 2012	Kidneys	* < 65 years	*97 kidneys transplanted
			*Witnessed CA	*19,6% PNF
			*No flow < 20 min	*60% 5 year graft survival
			*Resuscitation < 90 min after CA	
			*WIT < 135 min	
Lazzeri et al.,	Retrospective	25,	*15–65 years	Unknown
2020, Italy	June 2016–December 2018	Unknown	*Witnessed CA	
			*Relatives are present	
			*No flow < 20 min	
			*CA—hospital time < 90 min	
			*WIT < 150 min	
Fieux et al.,	Prospective	63,	*Absence neoplasia, systemic diseases	*43% effective donors
2009, France	February 2007–June 2008	Kidneys	*18—55 years	*31 kidneys transplanted
			*Witnessed and refractory CA	*90% 6 months graft survival
			*No flow < 30 min	
			*WIT < 150 min	
Champigneulle et al.	Prospective	126,	*Absence neoplasia, systemic diseases	*16,9% effective donors
2015, France	2010– 2012	Livers	*18–54 years	*11 livers transplanted
			*Refractory CA	*27% PNF
			*No flow < 15 min	*82% 1 year graft survival
			*WIT < 150 min	
Dupriez et al.,	Retrospective	39,	*Absence neoplasia, systemic diseases	*51% effective donors
2014, Belgium	1999–2014	Kidneys	* < 65 years	*25 kidneys transplanted
			*Witnessed and refractory CA	*5% PNF
			*No flow < 30 min	*86% 1 year graft survival
			*WIT < 120 min	

*CA* cardiac arrest, *PNF* primary non function, *WIT* warm ischemia time

#### Extracorporeal CPR and uDCD

In absence of ROSC, extracorporeal CPR (E-CPR) is one of the strategies to save lives. It is defined as the application of veno-arterial extracorporeal membrane oxygenation to provide circulatory support in patients in whom conventional CPR do not achieve ROSC [18, 38]. Current criteria proposed to start E-CPR are resumed in the Table 4 [11, 18, 39]. If E-CPR succeed in restoring cardiac activity, the overall survival depends on the selection of the patients, for starting E-CPR. The ARREST trial showed with strict criteria and late randomization in the CPR process, that E-CPR for patients with OHCA and refractory ventricular fibrillation significantly improved survival to hospital discharge and functional status compared with patients receiving ALS CPR [40]. Other larger studies have less convincing results with no significant differences though different inclusion and randomization strategies were used [41, 42]. Besides the use of E-CPR for treatment of refractory OHCA, it can also be a bridge to DBD and uDCD [13, 43]. However, 25% of the patients treated with E-CPR will be brain death, of whom 29–50% can become actual DBD [43–45]. Some patients undergo ECPR but never achieve ROSC and could potentially be eligible for uDCD with good graft outcomes [9, 11, 29, 46].

**Table 4:** Inclusion criteria for ECPR

Inclusion criteria for ECPR
Witnessed CA with bystander CPR
ALS CPR 5–15 min
Time establishing ECPR < 60 min from starting CPR
Age < 65–75 years
No major comorbidities
Known or suspected treatable cause of cardiac arrest

*ECPR* Extracorporeal cardiopulmonary resuscitation, *CA* cardiac arrest, *CPR* cardiopulmonary resuscitation

#### Ethical and economic implications

uDCD programs identifies ethical implications [47]. The first question is about the termination of resuscitation (TOR). There is a need of international guidelines to manage the TOR of patients in which E-CPR was initiated. Moreover, the effect of prolonged E-CPR on transplantable organs should be investigated. Secondly, obtaining the consent is a challenge and time is crucial for success of organ transplantation. In countries with opt-out system the assumption of consent is granted by law. In other jurisdiction, there is a need for strategies to facilitate the donation when asking to the family [13, 48]. Religious belief, education, economical status and miscommunication can also reduce implementation of an uDCD program. The International Liaison Committee on Resuscitation published a statement in 2023 with a review of the few data available on cost-effectiveness. Transplantation from deceased donors improve the cost effectiveness of OHCA [13]. Research is necessary to explore the role for E-CPR to facilitate uDCD on an ethical, physiological and economical level. A distinct difference between criteria for both purposes should be stated in hospitals who offer both modalities. The criteria for initiating E-CPR closely align with those for uDCD, making it essential to clearly document, once the decision to pursue a potential uDCD pathway is made, that this decision was made and reached in agreement with the treating team [13]. uDCD protocols require to reduce WIT and CIT as good as possible to assure good organ viability, prior to discussion with the family. Cardiac arrest patients could have criteria for uDCD and E-CPR, for that E-CPR should not be delayed if the patient is eligible to prevent the loss of a savable life [47].

## Conclusion

This review suggests that uDCD creates actual donations with acceptable outcomes for recipients when strict criteria, protocols, and preservation techniques are used. However, the process requires significant collaborative efforts from both the prehospital emergency team and the transplantation team, adhering to a strict timeframe within numerous critical decisions must be made by the teams, all while maintaining respect for the family.Reports from European databases support the implementation of such programs; however, they tend to overestimate the number of actual donors, as evidenced even by successful uDCD programs in Europe.

It is of utmost importance to increase the awareness of the possibility of uDCD programs for the (pre-hospital) emergency medical teams. Donation after uCDC is possible with acceptable results nonetheless more research is necessary to make an estimation about the cost and benefits, to phenotype the ideal donor and to establish guidelines about the use of E-CPR for organ donation and determine the limit of use for resuscitation.

## Data Availability

No datasets were generated or analysed during the current study.
